# Supervisors’ social dominance orientation, nation-based exchange relationships, and team-level outcomes

**DOI:** 10.3389/fpsyg.2022.865429

**Published:** 2022-10-26

**Authors:** Pegah Sajadi, Christian Vandenberghe

**Affiliations:** HEC Montréal, Université de Montréal, Montreal, QC, Canada

**Keywords:** social dominance orientation, national diversity, leader-member exchange differentiation, relationship conflict, team commitment

## Abstract

The prevalence of teams in contemporary organizations and the trend toward diversity in a workforce composed of members from multiple countries have drawn the attention of researchers on the consequences of diversity in workplaces. While there are potential benefits to diversity, relationship conflicts among team members may also result and affect team functioning. The aim of the present study was to explore how supervisors’ social dominance orientation, a tendency to support the arbitrary dominance of specific social groups over others, may relate to relationship conflicts and reduced team commitment within teams. A two-wave study in a sample of 931 individuals from 108 workgroups was conducted to examine the relationship between supervisors’ social dominance orientation and team functioning. Analyses indicated that supervisor social dominance orientation was associated with increased within-team differentiation of leader-member exchange (LMX) relationships based on team members’ national origin. Such LMX differentiation (LMXD) was related to more within-team relationship conflict and in turn to reduced collective team commitment. The implications of these findings for research on supervisor social dominance orientation, within-team nationality diversity, and team functioning are discussed.

## Introduction

The business trend toward globalization and the increasing percentage of non-native employees have made workforces across the world more diverse in terms of nationalities (Rosenauer et al., 2016; Homan et al., 2020). Indeed, the recent increase in remote working after the pandemic (Kniffin et al., 2021), which facilitates the formation of multinational workgroups in a virtual setting, suggests that studying the effects of nationality diversity has more relevance than ever. Nationality diversity is a mixed blessing for organizations (van Knippenberg et al., 2004). On the one hand, different beliefs, thinking styles, and ideas associated with nationality diversity may benefit diverse teams (Cox and Blake, 1991). On the other hand, teams with nationality diversity may suffer from some interpersonal problems such as relationship conflict (Ayub and Jehn, 2018)—interpersonal incompatibilities among group members which are often accompanied by tension and animosity among parties (Jehn et al., 1999). Researchers have extensively shown that relationship conflict is dysfunctional in teams (Jehn, 1995; De Dreu and Weingart, 2003; Somaraju et al., 2022). For instance, the meta-analytic study by De Dreu and Weingart (2003) reports a strong negative association between relationship conflict and team performance and member satisfaction. Also, Jehn (1995) has found a negative association between group members’ perceived relationship conflict and group members’ job satisfaction, liking of other group members, and their intent to remain in the group. Given these negative consequences, one would expect the team leaders to use practices that discourage relationship conflict in their teams. Yet, a close inspection of leaders’ behavior in organizations reveals that leaders themselves are sometimes responsible for creating relationship conflict in their workgroups (Zhao et al., 2019).

The present research seeks to understand *which* leaders (i.e., supervisors) promote relationship conflict in nationally diverse teams (i.e., teams with immigrants and native-born employees, which represents an important form of diversity) and through *which mechanisms* they do so. This study first draws from social dominance theory (Sidanius and Pratto, 2012) to suggest that supervisors’ social dominance orientation (SDO), i.e., the extent to which individuals desire and support the dominance of arbitrarily set dominant groups over subordinate groups (Pratto et al., 1994), leads them to differentiate among subordinates based on their national status (native-born vs. immigrant), a construct that is called nation-based leader-member exchange (LMX) differentiation (i.e., nation-based LMXD) in this study. According to LMX theory (Liden et al., 2006), supervisors build differential quality exchange relationships (ranging from low to high) with their subordinates. These exchange relationships have been shown to be differentiated within teams (Henderson et al., 2009). Such phenomenon, or LMXD, can be based on different factors (Chen et al., 2018; Han et al., 2021). Following this view, nation-based LMXD is defined as the tendency of supervisors to build higher quality LMX relationships with native-born subordinates compared to immigrant subordinates.

Second, drawing from faultline theory (Lau and Murnighan, 1998), this study further suggests that teams with higher levels of nation-based LMXD experience more relationship conflict. Faultlines are defined as “hypothetical dividing lines that may split a group into subgroups based on one or more attributes” (Lau and Murnighan, 1998, p. 328). Theory on faultlines posits that activated faultlines can create conflicting subgroups in diverse workgroups (Thatcher and Patel, 2011). Building on these core ideas, we argue that nation-based LMXD would promote relationship conflict by activating a nationality faultline. This study finally hypothesizes a negative association between relationship conflict and collective team commitment, a focal determinant of team performance (Mathieu and Gilson, 2012; Mahembe and Engelbrecht, 2013). The aim of the study was specifically to examine the proposed hypotheses within a large sample of employees and teams using data collected at two points in time. Of importance, the endogenous variables of the study measured at Time 2 (i.e., nation-based LMXD, relationship conflict, and team commitment) were controlled for their baseline level at Time 1.

This study contributes to the extant literature in at least three ways. First, this study contributes to the workforce diversity literature by examining the team-level processes and outcomes associated with nationality diversity. Although researchers have shown that diverse teams suffer from relationship conflicts (Pelled, 1996; Ayub and Jehn, 2006), to our knowledge, less research attention has been given to the role of supervisors in creating relationship conflict in diverse teams. This study shows that supervisors’ SDO, a specific individual difference variable related to how much supervisors endorse status differences among social groups, plays a critical role in developing nation-based LMXD, which ultimately promotes relationship conflict. Second, the present study contributes to the LMX literature, which has invested considerable effort in understanding the consequences of LMX differentiation. This study introduces nation-based LMXD as a novel and specific type of LMX differentiation that can emerge in diverse teams and documents its power to predict relationship conflict. Finally, this study contributes to enhance our understanding of the role of supervisor SDO and nation-based LMXD in affecting collective team commitment, thereby contributing to enlarge the array of determinants of team functioning in the modern workplace.

## Theoretical framework and hypotheses

### Nation-based leader-member exchange differentiation

Leader-member exchange theory (Graen and Uhl-Bien, 1995; Liden et al., 1997; Bauer and Erdogan, 2015), which has emerged as an important framework in the leadership literature, proposes that leaders build different types of exchange relationships with their subordinates (i.e., in-group and out-group exchanges; Dansereau et al., 1975) by treating some followers more favorably than others (Gerstner and Day, 1997). LMX differentiation (LMXD) is a concept that captures this differentiated treatment of subordinates by the leaders within teams (Maslyn and Uhl-Bien, 2005). As a result of LMXD, high LMX subordinates, compared to low LMX subordinates, would benefit from more advantages such as career progress (Wakabayashi and Graen, 1984; Wakabayashi et al., 1990; Scandura and Schriesheim, 1994), assignment of challenging jobs (Graen and Cashman, 1975), greater influence within the organization (Sparrowe and Liden, 2005), and receipt of more resources such as information and time (Dansereau et al., 1975).

Scholars have identified many factors that may explain why LMX differentiation occurs. These factors fall into individual (e.g., leadership style)-, team (e.g., aggressive culture)-, and organization (e.g., organizational structure)-level categories (Henderson et al., 2009). Due to one or more of these reasons, empirical studies indicate that LMX differentiation is very common in work groups; indeed, over 90% of work groups experience it (Dansereau et al., 1975; Liden and Graen, 1980), and it influences individual- and group-level outcomes. Such ubiquitous differentiation among subordinates can be based on the different factors.

The basis of LMX differentiation—those factors that determine the formation of differential LMX relationships between supervisors and their subordinates within a group (Chen et al., 2018)—has important individual- and group-level effects. For instance, Chen et al. (2018) introduced two bases for LMXD: members’ task performance and organizational citizenship behavior (OCB), and empirically showed how performance-based LMXD and OCB-based LMXD can alleviate the negative effects of LMX differentiation on group outcomes. Although LMX theorists have long theorized that for the sake of effectiveness and fairness, non-performance factors should not determine the quality of exchange relationships between a supervisor and his or her subordinates (Dansereau et al., 1975; Scandura, 1999), in reality, many non-performance factors may also influence LMX development such as liking, or demographic characteristics (Liden et al., 1993; Green et al., 1996; Randolph-Seng et al., 2016). Following this perspective, this study relies on the diversity literature and introduces national origin as a potential basis of LMXD in teams that are composed of native-born subordinates and foreign-born (i.e., immigrant) subordinates.

Immigrants, who are defined as people who are foreign-born but have the right to reside in their host country regardless of whether they have or do not have host country citizenship, are making a considerable share of the labor market and have attracted the attention of management scholars (Wrench, 2016). In 2020, immigrants accounted for more than 15% of the labor force of countries such as Germany and about 25% of the workforce in Australia, Canada, and New Zealand (Wrench, 2016). Much evidence indicates that immigrants experience unequal treatment in organizations (Foley et al., 2002; Bell et al., 2010; Enoksen, 2016; Villadsen and Wulff, 2018). This unequal treatment may be manifested in several ways. For example, immigrants may experience barriers to career advancement and be subject to jokes, negative comments, and stereotypes that demean their capabilities (Foley et al., 2002; Van Laer and Janssens, 2011; Ozturk and Berber, 2022).

This study draws from the above studies and suggest that immigrants may suffer from unequal treatment in terms of exchange relationships with their supervisors. The team-level construct of nation-based LMXD is proposed to reflect the extent to which team members perceive that the social exchange relationships between employees and supervisors are of a higher quality when employees are native-born (vs. immigrants). Thus, nation-based LMXD reflects whether LMX relationships are biased by the national origin of subordinates. In other words, the more the nation-based LMXD within a team, the more the distribution of LMX relationships would be based on national origin such that native-born subordinates would be favored over immigrants. In this manuscript, the focus is on perceived nation-based LMXD rather than on actual LMX configurations as LMX scholars have called for more subjective measures of LMX differentiation (Martin et al., 2018; Choi et al., 2020). This is because perceptions of the environment have typically more influence on job attitudes and behaviors compared to the objective reality (Kristof-Brown et al., 2005).

Acknowledging that the existence of diversity in a work team may not necessarily induce differential treatment (Lewis and Sherman, 2003; Sacco et al., 2003), one purpose of this study was to take a glimpse into the factors that may affect the emergence of nation-based LMXD in work teams composed of native-born and foreign-born employees. Specifically, the study focuses on supervisor SDO as a potential driver of nation-based LMXD as is discussed in the next section.

### Supervisor’s social dominance orientation and nation-based leader-member exchange differentiation

Social dominance theory (Pratto et al., 2006; Sidanius and Pratto, 2012) builds on sociological work on inequalities and social stratification (e.g., Lenski, 1984; Tilly, 1998) to examine the systems of group-based hierarchies in human societies. Within these hierarchies, those groups at the top (i.e., dominant groups) possess more social power and benefit from a disproportionate share of positive social value (e.g., wealth, high-status occupations, political power, better health care) while those at the bottom (i.e., subordinate groups) suffer from negative social value (e.g., substandard housing, underemployment, precarious work, and stigmatization) (Doane, 1997; Sidanius and Pratto, 2012). Beyond explaining how such hierarchies sustain over time, social dominance theory introduces an individual difference variable, namely SDO, which plays an important role in preserving these group-based hierarchies.

Social dominance orientation is a psychological component of social dominance theory that describes the tendency of an individual to believe in the legitimacy of predefined social structures and act in favor of sustaining inequality among social groups (Pratto et al., 1994). High SDO individuals prefer intergroup relations to be ordered along a dominant-subordinate continuum while low SDO individuals prefer intergroup relations to be equal (Pratto et al., 1994). SDO predicts many forms of group-based oppression such as racism, ethnocentrism, classism, and sexism (Sidanius and Pratto, 2012). Individuals high in SDO seek to reinforce inequality between groups to maintain their access to resources, such as power and wealth (Pratto and Shih, 2000). On the contrary, individuals with low SDO attach importance to egalitarianism and humanitarianism (Duckitt, 2001). While most studies of SDO come from the social psychology literature, there have been a number of recent studies conducted in organizational contexts that highlight the importance of SDO in predicting organizational behavior. Umphress et al. (2007), for example, found that as SDO increases, members of high-status groups find diverse organizations less attractive. Other research has shown that SDO is positively related to interpersonal deviance and negatively related to interpersonal citizenship (Shao et al., 2011). SDO is also positively linked to discrimination in hiring decisions and performance evaluations (Umphress et al., 2008; Simmons et al., 2015) as well as to abusive supervision (Khan et al., 2018).

Building on these studies, this study argues that if high SDO individuals have the authority to draw a hierarchy, they would be motivated to translate into reality the hierarchy they find legitimate, namely, a hierarchy that provides privileges to members of dominant groups. The differentiation of LMX relationships within teams is a hierarchy building process because, compared to low LMX subordinates, high LMX subordinates enjoy more advantages such as being more influential (Sparrowe and Liden, 2005), having more power to influence the group’s decisions (Scandura et al., 1986), and accessing more promotion opportunities (Wakabayashi and Graen, 1984). High LMX subordinates would thus benefit from more advantages than their low LMX counterparts, and supervisors may have a primary role in drawing this hierarchy. Supervisors may initiate high-quality exchange relationships with selected subordinates (Graen and Cashman, 1975) by offering their limited resources such as time and energy (Dansereau et al., 1975), and physical resources, interesting tasks, and valuable information (Graen and Cashman, 1975).

Extending the above argument to the context of teams composed of members from multiple nations, one may suspect that high SDO leaders, because they believe in the superiority of dominant social groups over subordinate social groups, will be likely to initiate higher quality exchange relationships with subordinates belonging to dominant groups and create a hierarchy of LMX relationships that brings benefits to the members of these groups. As in the hierarchy of social groups within host countries, immigrant groups are perceived to hold an inferior position compared to the dominant group of native-born citizens (Bauder, 2003; Reitz and Banerjee, 2007), immigrants may experience lower quality exchange relationships with supervisors who are high on SDO, reflecting some mistreatment based on national origin by high SDO supervisors. In support of this view, an empirical study by Costello and Hodson (2011) indicated that high SDO individuals tend to engage in prejudice against immigrants and resist to help them. Based on the above discussion, the following hypothesis is proposed:

***Hypothesis 1*****:** Supervisor SDO is positively associated with team level nation-based LMXD.

### Nation-based leader-member exchange differentiation and relationship conflict within teams

Workgroup diversity refers to the differences in workgroup members’ demographic attributes (e.g., ethnicity, gender, and age) or other characteristics (e.g., tenure, education, and professional background). These differences are associated with group members having different values, norms, beliefs, and worldviews that influence the way they define situations, see issues, and interact with others (see Alderfer, 1987; Ely and Thomas, 2001). As a result of such differences, diverse workgroups may be more creative (Cox and Blake, 1991). However, these groups may also experience more conflict depending on the nature of the differences across group members and the ability to manage these differences, and on the potential influence of factors from the larger environment in which they are embedded (Alderfer, 1987; Jehn, 1995; Pelled et al., 1999; Ayub and Jehn, 2018).

The difficulty to deal with the consequences of team composition diversity may also be amplified by faultlines. Faultlines are hypothetical lines of division that breakup a workgroup into relatively homogeneous subgroups based on the diversity attributes of group members (Lau and Murnighan, 1998). For instance, the national origin faultline may divide groups into immigrant and native-born subgroups. According to Lau and Murnighan (1998), activated faultlines in diverse groups exacerbate the impact of diversity and augment the likelihood that members perceive subgroups to exist and experience subgroup conflict. Activated faultlines divide workgroups into conflicting subgroups in which members define themselves as part of these subgroups rather than as part of the larger group. Faultlines generally exist when the group members perceive that subgroups emerge from the divides on demographic characteristics (e.g., age, gender, etc.) (Jehn and Bezrukova, 2010). The activation process for faultlines can be triggered by different factors including *differential treatment* of employees based on their demographic characteristics, for instance, when resources or punishments are differentially distributed across different demographic groups (Chrobot-Mason et al., 2009).

Following the above logic, this study argues that nation-based LMXD contributes to the activation of a nationality faultline within work teams composed of native-born vs. foreign-born employees. This is because, by building nation-based LMX differentiation within the team, supervisors would differentially treat native-born and immigrant subordinates and thus would activate a nationality faultline, which in turn would increase the likelihood of emergence of within-team relationship conflict. Moreover, as it was previously argued that nation-based LMXD is namely driven by supervisor SDO, this study posits that supervisor SDO will indirectly relate to more within-team relationship conflict through increased nation-based LMXD. Thus, the following hypotheses are proposed.

***Hypothesis 2*****:** Team-level nation-based LMXD is positively associated with within-team relationship conflict.

***Hypothesis 3*****:** Team-level nation-based LMXD mediates a positive relationship between supervisor SDO and within-team relationship conflict.

### Within-team relationship conflict and collective team commitment

It can be expected that the occurrence of more within-team relationship conflicts as induced by higher nation-based LMXD will then result in reduced collective team commitment. Following Klein et al. (2012, 2014) reconceptualization of employee commitment, commitment can be defined as “a volitional psychological bond reflecting dedication to and responsibility for a particular target” (Klein et al., 2012, p. 137). This proposed definition makes commitment amenable to application to any target of relevance in the workplace, with this approach having received consistent empirical support (Klein et al., 2014). From an empirical perspective, Klein et al.’s (2014) unidimensional, target-free measure (KUT) of commitment has been found to be strongly positively related to the measure of affective commitment developed by Meyer et al. (1993). From a conceptual perspective, (Meyer and Herscovitch, 2001, p. 301) have defined commitment as “a force that binds an individual to a course of action of relevance to one or more targets” and have suggested that in the case of affective commitment, the mindset that accompanies this force is the desire to pursue a course of action in favor of the target. Given the empirical closeness between the KUT and affective commitment, the previous commitment literature, which has largely examined the role of affective commitment in the workplace, remains a relevant source of reference, even when commitment is measured through the KUT as is done in the present study (Vandenberghe, 2021).

Given this study’s focus on supervisor SDO and within-team nation-based LMXD and relationship conflict, the relations between these constructs and team commitment or team members’ attachment to their team (Gardner et al., 2011), which is a major outcome and indicator of team functioning (Mathieu et al., 2008), will be examined. At the team level, when members consistently perceive that relationship conflict exits among team members, they are unlikely to share a sense of membership in and attachment to the team as a whole. This is because teams with relationship conflicts suffer from destructive team processes including the lack of trust (Langfred, 2007) and cohesion (Jehn and Mannix, 2001). Indeed, relationship conflict surfaces as an increase in expression of negative emotions (Thiel et al., 2019). These negative emergent states accompanying the emergence of within-team relationship conflict are likely to jeopardize team members’ collective commitment to their team. Although, to our knowledge, the team-level association between relationship conflict and team commitment has not been examined, researchers have consistently reported a negative association between relationship conflict and affective commitment at the individual level (Thomas et al., 2005; Lee et al., 2018). By extension, this study argues that within-team relationship conflict will be related to lower collective team commitment. Moreover, as it was previously argued that nation-based LMXD would relate to more within-team relationship conflict, the former is expected to be indirectly related to reduced collective team commitment through increased within-team nation-based LMXD. Thus, the following, remaining hypotheses are proposed.

***Hypothesis 4*****:** Within-team relationship conflict negatively relates to team-level commitment to the team.

***Hypothesis 5*****:** Within-team relationship conflict mediates a negative relationship between team-level nation-based LMXD and team-level commitment to the team.

## Materials and methods

### Sample and procedure

**FIGURE 1:**
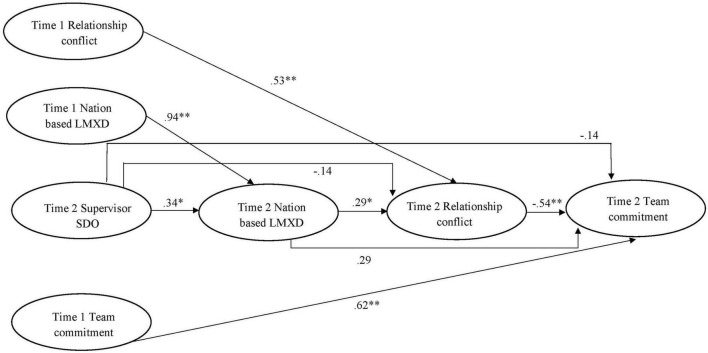
Structural equation modeling results for the hypothesized model. For the sake of clarity, control variables (team size, supervisor place of birth, and LMXD) are omitted. *p < 0.05; ^**^p < 0.01.

Data were collected at two points in time from employees in eight governmental organizations located in the Quebec province, Canada. The first wave of the data collection took place between September and November 2020 while the second wave was set between April and July 2021. Upon the agreement of the organizations’ human resource management directors, prospective participants were contacted by email to participate in a multi-wave study of job attitudes. An introductory message advised respondents that participation was voluntary, and responses would remain confidential. The criteria for participation were having (a) salaried employment and (b) an identifiable supervisor. Although the questionnaires could be completed in French or English, all respondents chose to complete the French version of the questionnaires. To match responses across measurement times, a unique code was assigned to each participant. At Time 1, employees completed demographic questions while at Time 2, they were surveyed about LMX (refer to control variables section). At Time 1 and Time 2, employees were surveyed about nation-based LMXD, relationship conflict, and team commitment, while supervisor SDO was self-reported by supervisors at Time 2. Data on the control variables of supervisor place of birth (Time 1) and team size (Time 1) were obtained from supervisors (refer to control variables section). Employee data were then aggregated at the team level and combined with supervisor SDO to conduct the analyses related to this research model (Figure 1). Time 1 employee data on nation-based LMXD, relationship conflict, and team commitment served as baseline controls when testing the hypotheses at the team level, which involved Time 2 data. This approach is an efficient way by which common method variance can be mitigated in data analyses (Maxwell and Cole, 2007).

Dropping those participants who failed the attention check item (Huang et al., 2015), 1,104 usable responses at Time 1 and 1,356 usable responses at Time 2 were received. The difference in sample size between Time 1 and Time 2 is due to new employees being recruited and added in the participating organizations between the two survey times. Matched data across time were available for 931 employees affiliated with 108 work teams. The average age of these employees was 48 years (*SD* = 11.06), their average organizational tenure was 9 years (*SD* = 9.22), 36% were male, and 25% were born outside of Canada. As 173 of the 1,104 Time 1, participants did not complete the Time 2 survey, an attrition analysis through logistic regression was conducted to determine whether there was a systematic attrition bias between Time 1 and Time 2. Specifically, a dichotomous variable reflecting Time 2 attrition (i.e., 0 = Time 1 respondents who completed the Time 2 survey vs. 1 = those who dropped out at Time 2) was regressed onto nation-based LMXD (*b* = −0.07, *SE* = 0.11, *ns*), relationship conflict (*b* = 0.11, *SE* = 0.11, *ns*), and team commitment (*b* = −0.03, *SE* = 0.06, *ns*) from Time 1. These non-significant results indicate there was no attrition bias among respondents between Time 1 and Time 2.

### Measures

#### Social dominance orientation

Social dominance orientation was measured using Sidanius et al.’s (1996) 16-item scale. Based on an exploratory factor analysis of the items, which identified a single factor, the 9 items with the highest loadings (>0.40) were retained. Sample items from the 9-item reduced scale are “To get ahead in life, it is sometimes necessary to step on other groups” and “No one group should dominate in society” (reverse coded). Responses were rated on a 7-point scale ranging from 1 (*strongly disagree*) to 7 (*strongly agree*). This scale had an internal consistency of 0.92.

#### Nation-based leader-member exchange differentiation

The perception of nation-based LMXD was measured with six items adapted from Choi et al. (2020). These items, which measured perceptions of LMXD, were adapted by incorporating national origin as the basis for LMXD. The six items are “Native-born members have a better relationship with my manager than immigrants”; “My manager treats native-born members better than immigrants”; “My manager is more loyal to native-born members compared with immigrants”; “Relative to the immigrants in my workgroup, native-born members receive more support from my manager”; “My manager seems to like native-born members more than immigrants”; and “My manager respects native-born members more than immigrants.” Responses were rated on a 7-point scale ranging from 1 (*strongly disagree*) to 7 (*strongly agree*). This scale demonstrated high internal consistency at Time 1 (α = 0.96) and Time 2 (α = 0.98).

#### Relationship conflict

Perception of relationship conflict among team members was measured using a three-item measure developed by Jehn and Mannix (2001). A sample item is “How much relationship tension is there in your work group?” Responses were rated on a 7-point scale ranging from 1 (*strongly disagree*) to 7 (*strongly agree*). This scale had high reliability at Time 1 (α = 0.92) and Time 2 (α = 0.92).

#### Team commitment

The four-item KUT scale developed by Klein et al. (2014) was used to measure team commitment. The four items referred to the work team as the target of commitment. A typical item was “To what extent do you care about your work team?” Responses were rated on a 5-point scale ranging from 1 (*not at all*) to 5 (*extremely*). The alpha reliability was 0.93 at both Time 1 and Time 2.

#### Control variables

Following Spector and Brannick’s (2011) recommendations to include control variables that may influence hypothesized relationships, several relevant variables were controlled for in testing hypotheses. First, the baseline (i.e., Time 1) levels of nation-based LMXD, relationship conflict, and team commitment were controlled for. Second, within-team differentiation on LMX relationships, as a potential predictor of relationship conflict and team commitment, was controlled for. LMX was rated by the employees at Time 2 using the 12-item LMX-MDM scale from Liden and Maslyn (1998). A typical item is “I like my supervisor very much as a person” (α = 0.94). Consistent with previous studies conducted at the group level (e.g., Nishii and Mayer, 2009), the amount of LMX differentiation was assessed by calculating the within-team variance (measured by *SD*) on LMX scores. Furthermore, supervisor place of birth (1 = Canada; 2 = outside of Canada; Time 1) was controlled for as research suggests that it may influence LMX distribution in diverse teams (Pichler et al., 2019). Finally, team size (Time 1), as a potential predictor of relationship conflict and team commitment, was controlled for.

### Research design and statistical analysis

As illustrated in Figure 1, this study used a team-level design to explore how supervisor SDO affected nation-based LMXD, which in turn was thought to influence team relational conflict and ultimately team goal commitment. As all these constructs were measured at the same time, we controlled for the baseline levels of the endogenous variables (i.e., nation-based LMXD, relationship conflict, and team commitment) to obtain a more robust assessment of the hypothesized relationships. This resulted in a complex design where all the paths among the constructs measured at Time 2 were estimated while controlling for the autoregressive effects of Time 1 nation-based LMXD, relationship conflict, and team commitment (Figure 1). This study employed Mplus 7.4 (Muthén and Muthén, 2012) for statistical analyses. First, since this study involved team-level constructs (Figure 1), the appropriateness of aggregating individual responses to scale items to the team level was examined. Second, a series of multilevel confirmatory factor analyses (CFAs) were conducted to examine whether the focal constructs were distinguishable. Third, as the theoretical model controlled for Time 1 nation-based LMXD, relationship conflict, and team commitment, measurement invariance across time was tested to ensure that the constructs’ meaning remained stable (Cole and Maxwell, 2003; Millsap, 2012). Next, the descriptive statistics for the variables of interest and the bivariate associations among them were obtained. The hypotheses were tested through two-stage multilevel structural equation modeling (MSEM; Heck and Thomas, 1999) using full information maximum likelihood (FIML) estimation *via* Mplus 7.4 (Muthén and Muthén, 2012). The indirect effects were examined using a bootstrapping approach (Preacher and Hayes, 2008) on the team-level model^1^ and bias-corrected confidence intervals (CIs) obtained from 10,000 bootstrapped samples.

## Results

### Data aggregation at the team level

The opportunity to aggregate individual responses to nation-based LMXD, relationship conflict, and team commitment to the team level was examined by calculating within-team agreement through the interrater agreement index [r_wg(j)_; James et al., 1984] and ICC(1) and ICC(2) intraclass correlations (LeBreton and Senter, 2008). The median values for r_wg(j)_ were sizeable for nation-based LMXD (0.99), relationship conflict (0.76), and team commitment (0.82), indicating strong within-team agreement on these variables. Similarly, the ICC(1) values for nation-based LMXD (0.14), relationship conflict (0.24), and team commitment (0.08) indicated meaningful variance on scale scores across teams (LeBreton and Senter, 2008). Finally, the ICC(2) values for nation-based LMXD (0.57), relationship conflict (0.72), and team commitment (0.45) provided evidence of acceptable reliability of team-level scores on the variables of interest (LeBreton and Senter, 2008). These results suggest that individual data could be aggregated at the team level.

### Confirmatory factor analyses

A series of multilevel confirmatory factor analyses (CFAs) was conducted in Mplus 7.4 (Muthén and Muthén, 2012). In terms of fit indices, the chi-square (χ^2^) test, which is known as a test of exact fit, was used, as well as the comparative fit index (CFI), the Tucker–Lewis index (TLI), the root mean square error of approximation (RMSEA), and the standardized root mean square residual (SRMR) as other fit indices. For the CFI and TLI, values exceeding 0.90 and 0.95 are considered as the indications of adequate and excellent fit, respectively, values below 0.06 for the RMSEA indicate excellent fit, and values below 0.08 for the SRMR indicate good fit (Hu and Bentler, 1999). First, a CFA model, in which nation-based LMXD, relationship conflict, and team commitment were treated as both within-team and between-team factors while supervisor SDO was treated as a between-team factor, was tested. The results of the CFA revealed that some SDO items had a factor loading below the conventional cutoff value (0.40). We dropped these items and used the remaining nine items for the SDO measure. The revised CFA model fitted the data well, [χ^2^(818) = 1351.84, *p* < 0.001, CFI = 0.98, TLI = 0.98, RMSEA = 0.02, SRMR_within_ = 0.02, SRMR_between_ = 0.17 (note that although the value for SRMR_between_ was large, the general profile of the fit indices suggests good fit of the model (Hu and Bentler, 1999))]. This model yielded a better fit than three alternative, more parsimonious models: (a) a model in which nation-based LMXD and relationship conflict items loaded on a single factor at Time 1 and Time 2, Δχ^2^(20) = 4333.35, *p* < 0.001; (b) a model specifying all three parallel variables from Time 1 and Time 2 to merge into a single set of three factors, Δχ^2^(27) = 5851.30, *p* < 0.001; and (c) a one-factor model where all items loaded on a single factor, Δχ^2^(35) = 14906.45, *p* < 0.001. These results indicate that the study variables were discriminant.

### Measurement invariance

To examine the measurement invariance over time of the three constructs measured at Time 1 and Time 2 (i.e., nation-based LMXD, relationship conflict, and team commitment), a sequential approach was adopted where increasingly stringent constraints were added to the CFA model (e.g., Vandenberg and Lance, 2000). The baseline model was a configural model (i.e., equality of factor structure), and the next models were weak, strong, and strict invariance models, reflecting a sequence of increasingly stringent equality constraints on factor loadings, thresholds, and uniquenesses, respectively. Robust maximum likelihood (MLR) was used to examine measurement invariance. The results are reported in Table 1. As can be seen, the Δχ^2^ values remained non-significant along the sequence of models with increasing constraints of equality (from configural invariance to strict invariance). These results support strict invariance among the constructs and stable psychometric properties across time (Byrne et al., 1989; Cheung and Lau, 2012).

**TABLE 1:** Tests of measurement invariance across time.

Model	χ^2^	*df*	CFI	TLI	RMSEA	Δχ^2^	Δ*df*
Configural invariant model	387.82*	268	0.99	0.98	0.02	-	-
Weak invariant model (loadings)	406.01***	278	0.99	0.98	0.02	18.19	10
Strong invariant model (loadings, thresholds)	423.26***	288	0.99	0.98	0.02	17.25	10
Strict invariant model (loadings, thresholds, uniquenesses)	449.67***	291	0.98	0.98	0.02	26.41	3

Full information maximum likelihood estimation was used. df, degrees of freedom; CFI, comparative fit index; TLI, Tucker–Lewis index; RMSEA, root mean square error of approximation.

*p < 0.05; ***p < 0.001.

### Descriptive statistics and correlations

Descriptive statistics, correlations, and reliability coefficients are reported in Table 2. Supervisor SDO was positively related to Time 2 nation-based LMXD (*r* = 0.29, *p* < 0.10). Time 2 nation-based LMXD was positively related to Time 2 relationship conflict (*r* = 0.30, *p* < 0.05) while the latter was negatively related to Time 2 team commitment (*r* = −0.66, *p* < 0.01).

**TABLE 2:** Descriptive statistics and correlations among study variables.

Variable	M	SD	1	2	3	4	5	6	7	8	9
**Individual level**											
1. Nation-based LMXD (T1)	1.25	0.95	(0.96)								
2. Nation-based LMXD (T2)	1.28	0.84	0.69**	(0.98)							
3. Team commitment (T1)	4.04	0.65	−0.17**	−0.12**	(0.93)						
4. Team commitment (T2)	4.05	0.62	−0.12**	−0.14**	0.63**	(0.93)					
5. Relationship conflict (T1)	2.32	1.17	0.26**	0.26**	−0.24**	−0.19**	(0.92)				
6. Relationship conflict (T2)	2.11	1.11	0.16**	0.28**	−0.16**	−0.24**	0.61**	(0.92)			
**Team level**											
1. Supervisor SDO (T2)	1.64	0.12	(0.92)								
2. Nation-based LMXD (T1)	1.22	0.12	–0.06								
3. Nation-based LMXD (T2)	1.30	0.19	0.29+	0.89**							
4. Team commitment (T1)	4.09	0.10	–0.04	–0.07	–0.07						
5. Team commitment (T2)	4.45	0.20	–0.03	–0.18	–0.19	0.56+					
6. Relationship conflict (T1)	2.33	0.72	0.00	0.00	0.00	0.00	–0.35				
7. Relationship conflict (T2)	2.10	0.67	–0.05	0.31*	0.30*	–0.02	−0.66**	0.59**			
8. LMXD (T2)	0.64	0.29	0.00	0.00	0.00	0.00	−0.59**	0.07*	0.40**		
9. Team size (T1)	9.11	4.77	0.00	0.00	0.00	0.00	0.01	0.11	0.08	0.26**	
10. Supervisor place of birth (T1)	1.11	0.31	0.00	0.00	0.01	0.00	–0.01	0.05	0.03	–0.01	0.03

T1, Time 1; T2, Time 2; LMXD, leader-member exchange differentiation; SDO, social dominance orientation; for supervisor place of birth: 1, Canada; 2, outside of Canada. Alpha reliabilities at the individual level (including for supervisor SDO) are listed within parentheses along the diagonal.

+*p* < 0.10; *p < 0.05; **p < 0.01.

### Hypothesis testing

The hypothesized model yielded a good fit to the data: χ^2^(755) = 1483.14, *p* < 0.01, CFI = 0.97, TLI = 0.97, RMSEA = 0.03, SRMR_within_ = 0.06, SRMR_between_ = 0.22. Hypothesis 1 predicted that the higher the supervisor’s SDO, the higher the team’s level of nation-based LMXD. As shown in Table 3, controlling for Time 1 nation-based LMXD, supervisor SDO was significantly positively related to Time 2 nation-based LMXD (β = 0.34, *SE* = 0.18, *p* < 0.05). Therefore, Hypothesis 1 is supported. Hypothesis 2 posited that teams higher on nation-based LMXD would experience more relationship conflict. As shown in Table 3, controlling for Time 1 relationship conflict, nation-based LMXD had a significant and positive association with Time 2 relationship conflict (β = 0.29, *SE* = 0.14, *p* < 0.05), thereby providing support to Hypothesis 2. Finally, Hypothesis 4 predicted that teams with more relationship conflict would display lower team commitment. As shown in Table 3, controlling for Time 1 team commitment, relationship conflict was significantly negatively related to Time 2 team commitment (β = −0.54, *SE* = 0.14, *p* < 0.01). Therefore, Hypothesis 4 is supported.

**TABLE 3:** Structural equation model analysis for hypothesized model: Structural parameter estimates.

	Nation-based LMXD (T2)		Relationship conflict (T2)		Team commitment (T2)	
			
Variable	β	SE	β	SE	β	SE
Team size (T1)			–0.05	0.08	0.11	0.11
Supervisor place of birth (T1)	0.01	0.15	0.01	0.08	0.07	0.11
LMXD (T2)			0.32**	0.07	−0.40**	0.13
Supervisor SDO (T2)	0.34*	0.18	–0.14	0.09	–0.14	0.12
Nation-based LMXD (T1)	0.94**	0.20				
Nation-based LMXD (T2)			0.29*	0.14	0.29	0.18
Relationship Conflict (T1)			0.53**	0.06		
Relationship Conflict (T2)					−0.54**	0.14
Team commitment (T1)					0.62**	0.19
Team commitment (T2)						
*R* ^2^	0.96*	0.39	0.51**	0.08	0.91**	0.18

T1, Time 1; T2, Time 2; LMXD, leader-member exchange differentiation; SDO, social dominance orientation; for supervisor place of birth: 1, Canada; 2, outside of Canada.

*p < 0.05; **p < 0.01.

The bootstrapping method was employed to test the significance of the indirect relations in the model. Table 4 presents the CIs for the hypothesized indirect relations, as well as the total effects. As can be seen from this table, the relationship between supervisor SDO and relationship conflict through nation-based LMXD was positive but non-significant (0.02, 95% CI [−0.03, 0.09]) as the bootstrap CI contained zero. Thus, Hypothesis 3 is not supported. Finally, Hypothesis 5 stated that nation-based LMXD would be indirectly related to team commitment through within-team relationship conflict. As shown in Table 4, the relationship between nation-based LMXD and team commitment through relationship conflict was significantly negative (−0.05, 95% CI [−0.12, −0.01]) as the CI did not include zero. Thus, Hypothesis 5 is supported.

**TABLE 4:** Summary of mediation analyses using 10,000 bootstrap samples.

		95% CI	
		
	Estimate	LB	UB
**Total effects**			
SDO → Relationship conflict (Time 2)	–0.05	−0.16	0.08
Nation-based LMXD → Team commitment (Time 2)	0.06	−0.09	0.20
**Specific indirect effects**			
SDO → Nation-based LMXD → Relationship conflict (Time 2)	0.02	−0.03	0.09
Nation-based LMXD → Relationship conflict → Team commitment (Time 2)	−0.05*	−0.12	–0.01

CI, confidence interval; LB, lower bound; UB, upper bound. Estimates of total and indirect effects are based on the final structural equation model displayed in Figure 1.

*p < 0.05.

## Discussion

### Implications for theory development

The findings of this study provide a number of new insights into the role of supervisors in shaping team functioning and outcomes among teams composed of members from multiple nations. Scholars have studied the role of leadership in diverse workgroups and have introduced leadership styles (e.g., transformational leadership; Wang et al., 2013), practices (e.g., inclusive leadership; Leroy et al., 2022), and competencies (e.g., communication competencies; Lu et al., 2021) that may help diverse teams harvest the benefits of diversity. However, this line of research has scarcely examined the potential negative role that supervisors may play in diverse teams. The present study looked at this negative role through the lens of supervisor SDO. Previous research has indicated that SDO positively relates to interpersonal deviance (Shao et al., 2011) and abusive supervision (Khan et al., 2018) and is positively associated with discrimination in hiring decisions (Umphress et al., 2008; Simmons et al., 2015). This study extends this line of work by providing empirical evidence that within work teams including members from diverse nations, high SDO supervisors tend to engage in LMX relationships of a higher quality with native-born subordinates and LMX relationships of lower quality with foreign-born subordinates. This in turn was found to be associated with within-team relationship conflict. Moreover, within-team relationship conflict was associated with collective team commitment. Note, however, that the indirect relationship between supervisor SDO and within-team relationship conflict through nation-based LMXD was non-significant, which may be due to a lack of power or to the fact that baseline levels of the mediator and outcome variable were controlled for, hence making this test more stringent. Although the relation between supervisor SDO and nation-based LMXD was significant in the sample of this study, the sample provided a likely conservative test of the importance of supervisor SDO because of the low mean of SDO in the sample (i.e., 1.64/5). According to the research commissioned by Forbes Insights and conducted by Oxford Economics, which provides a unique ranking of employee diversity across fifty global economies, Canada is among the most diverse countries in the world. Canada’s high score on the Migration Integration Policy Index (MIPEX) and low score on the Fragile States Index (FSI) also indicate that Canada has conceived of itself as one of the best immigrant-friendly countries. The low mean on supervisor SDO in the sample may reflect the fact that Canada has a diversified workforce where immigrants are relatively well-perceived and integrated (Fischer et al., 2012). Future research should examine the consequences of supervisors’ SDO in contexts and countries where systemic inequality, competition, and resource-based threat are higher as these factors heighten the level of SDO among individuals (Cohrs and Stelzl, 2010).

Future research is also warranted to explore the potential moderators that can buffer the negative relation between supervisor SDO and team processes and outcomes. For example, it might be that policies and practices that discourage discriminatory behaviors among managers and facilitate the emergence of work climates that foster inclusion of immigrants may reduce the negative association between supervisor SDO and LMX faultlines and curb the salience of subgroups of employees based on their national origin. Following this view, high SDO supervisors would be more likely to engage in differential LMX relationships with subordinates based on their national origin when they are affiliated with organizations displaying less inclusive climates.

Furthermore, the present study is the first to examine the role of a non-performance basis for LMXD perceptions in work teams. A new approach to LMXD, labeled nation-based LMXD, was developed that captures the extent to which LMX relationships associated with the supervisor are driven by a national origin faultline. The six-item scale, which was adapted from Choi et al. (2020), was found to be a reliable measure of nation-based LMXD that was independent from the dispersion of LMX relationships within teams (i.e., LMXD). It is also worth noting that the relation between nation-based LMXD and within-team relationship conflict was incremental to LMXD *per se*. This denotes the power of this variable in relating to important team outcomes. For further exploration in future research, it would be interesting to explore what other team-level outcomes might be affected by nation-based LMXD. Valuable outcomes for this work might be team cohesion and team performance. One may also speculate that nation-based LMXD may differentially influence subordinates from immigrant groups compared to native-born subordinates because native-born subordinates, who tend to receive better treatment owing to their status as members of a dominant social group, should feel more comfortable with nation-based LMXD. Future research may also consider subordinates’ own level of SDO as this may also play an important role in reactions to nation-based LMXD. Low SDO subordinates, because they do not believe in the legitimacy of a hierarchy among social groups, may be more negatively influenced by exposure to nation-based LMXD than high SDO subordinates. Future research can thus explore the differential consequences of nation-based LMXD among subordinates with different levels of SDO.

This study also contributes to the diversity literature by adding to the growing body of research that examines the downside of diversity (e.g., relationship conflict) in work teams. Scholars have often used insights from research on social categorization and intergroup relations to predict that differences between people elicit social categorization processes, which in turn disrupt group functioning and promote competition and conflict among employees. However, as van Knippenberg and Haslam (2003) argue, it is intergroup prejudice and bias that may disrupt group processes, not categorization *per se*. This study supports this view as, in *post-hoc* analyses, the magnitude of diversity indicators did not contribute significantly to group outcomes.^2^ Yet, the findings indicated that one individual difference variable, namely, supervisor SDO, which is known to foster intergroup prejudice, was detrimental to team-level outcomes.

### Practical implications

This study also has practical implications for work teams with members with diverse backgrounds. It underscores that paying attention to the characteristics of candidates for leadership positions in a diverse environment is important since the roots of relationship conflict may partly reside in supervisors’ characteristics (i.e., SDO). An effective strategy to reduce interpersonal tensions in diverse groups would be to ensure that individuals in leadership positions do preferably display low levels of SDO. Indeed, top managers may more easily promote inclusive climates if they hold low levels of SDO, and this would pave the way to influencing employees’ SDO itself. SDO develops from several factors, including socialization experiences, social context, and individual temperament (e.g., empathy, aggression) (Sidanius et al., 2004). For instance, SDO tends to be higher in dominant social groups (Sidanius and Pratto, 2012). As research suggests that transformational leadership promotes inclusive climates (Kearney and Gebert, 2009), organizations with diverse workgroups may be well-advised to appoint leaders with a transformational leadership style or to train them to develop transformational skills, so that employees’ own SDO levels could decrease over time in such inclusive climate.

### Strengths and limitations

As any study, this research has limitations. First, all measures were self-reported, making the findings susceptible to be affected by common method variance (Podsakoff et al., 2012). However, some features of the research design and data analyses provide some confidence in the robustness of the results. On the one hand, while within-team LMXD and relationship conflict and collective team commitment were assessed by subordinates, supervisor SDO was reported by supervisors themselves, so that the study was basically multi-source. Moreover, while examining the relation between supervisor SDO and nation-based LMXD, the dispersion of LMX relationships within teams was controlled for. Thus, the relation between supervisor SDO and nation-based LMXD was unique, independently from LMX relationships. On the other hand, this study controlled for the baseline (i.e., Time 1) levels of all endogenous variables (i.e., nation-based LMXD, within-team relationship conflict, and collective team commitment), thus considerably reducing any endogeneity related to the findings (Podsakoff et al., 2003) and lending confidence in their robustness. Second, despite the strengths of the design and analyses, one cannot conclude to causal relationships among the constructs. For example, it might be that team members with higher levels of team commitment perceive fewer relationships conflicts and ultimately less differentiation of LMX relationships based on the national origin as the members. Further research using fully cross-lagged designs is warranted to clarify temporal relationships among the constructs. Third, from a theoretical perspective, it would be worth exploring how other leadership models such as servant leadership could influence the findings reported in the present study. For example, even though supervisor SDO was related to high LMX differentiation based on nationality diversity, this relation may be tempered if at the same time the supervisor adopts servant leadership practices that make employees feel supported (Hu et al., 2020). Future research could explore that possibility. Fourth, the data from this study were obtained from government agencies located in the Quebec province, Canada. Therefore, both the nature of jobs (civil servants) and language might limit the generalizability of the findings to other workplaces and countries. Finally, this study was based on a large sample of 931 employees pertaining to 108 teams and the analyses were conducted at the team level as justified by appropriate aggregation statistics. Therefore, the limitations regarding causal connections among the variables are counterbalanced by the fact that this study captured phenomena that reliably reflected team level processes.

## Conclusion

The present study examined a model of the antecedent and outcome variables of differential LMX relationships among work teams composed of members from diverse national origins. Based on a sample of 108 work teams from eight Canadian organizations, this study indicates that supervisors’ SDO relates positively to nation-based LMXD, which in turn relates to more within-team relationship conflict. In turn, relationship conflict relates to lower collective team commitment. As such, this study highlights how the social dominance beliefs of leaders can be associated with diverse malfunctions within teams where subordinates from diverse national origins work together in the pursuit of team goals. Given these findings, further attempts at exploring other leadership and work-related factors as antecedents of nation-based LMXD and how these factors may ultimately affect team functioning are warranted.

## Data availability statement

The raw data supporting the conclusions of this article will be made available by the authors, without undue reservation.

## Ethics statement

The studies involving human participants were reviewed and approved by Comité d’éthique de la recherche (HEC Montréal). The patients/participants provided their written informed consent to participate in this study.

## Author contributions

PS performed the statistical analyses and took the lead for writing the related sections. CV contributed to the acquisition of data. Both authors contributed to the design, revision, improvement of the manuscript, interpretation of the results, wrote the manuscript’s theoretical introduction, methods, results, and discussion, and approved the submitted version.

## Footnotes

1 The team level model is based on group-level averages of individual scores on the variables.

2 For exploratory purposes, we checked whether ethnicity diversity, nation-based diversity, and religion diversity would affect the results associated with our model. We thus examined a model where these controls were included. As the effects of these variables were non-significant, we dropped them from the model.
